# Where and how do mammalian cells shape autophagosomes?

**DOI:** 10.1016/j.jbc.2026.111428

**Published:** 2026-04-07

**Authors:** Claudia Puri, David C. Rubinsztein

**Affiliations:** 1 Cambridge Institute for Medical Research, The Keith Peters Building Cambridge, Cambridge Biomedical Campus, Cambridge, UK; 2 UK Dementia Research Institute, The Keith Peters Building Cambridge, Cambridge Biomedical Campus, Cambridge, UK

**Keywords:** Alzheimer's disease, autophagy, endoplasmic reticulum, RAB11A, recycling endosome

## Abstract

Autophagy is a fundamental cellular process responsible for degrading and recycling cytoplasmic components and regulates homeostasis, development, and survival under stress. Autophagy plays critical roles in diseases, including neurodegeneration, cancers, and various infectious and inflammatory conditions. While the molecular machinery of autophagy has been well studied, increasing evidence highlights a complex interplay between autophagy and endocytosis. Traditionally, mammalian autophagosomes were believed to originate from compartments closely associated with the endoplasmic reticulum or the endoplasmic reticulum itself. However, more recent research has demonstrated that the recycling endosome (RE) serves as the main platform for autophagosome formation. The recruitment of WIPI2, an essential autophagy protein, to autophagosome initiation sites depends on its coincident detection of phosphatidylinositol 3-phosphate and RAB11A, an RE marker. This enables conjugation of LC3 (microtubule-associated protein light-chain 3) family members to the RE membranes to become nascent autophagosomes. These findings underscore the critical role of RAB11 compartment in autophagosome biogenesis. Contrary to the conventional model that has inferred that autophagosomes derive from spherical precursors with single apertures, structured illumination microscopy reveals these precursors are finger-like structures—much like a hand grasping an object. We will describe the experimental path that led to an understanding of how autophagosomes form from outgrowths of the REs, then close after engulfing their contents. This step is a prerequisite for the final step of autophagosome formation, the release of autophagosomes from the RE membranes, a process that is perturbed by a major Alzheimer’s disease gene.

Macroautophagy (hereafter autophagy) is a highly conserved intracellular pathway that sequesters cytosolic material, misfolded proteins, and damaged organelles in double-membrane structures called autophagosomes before delivering them to lysosomes for degradation. The term “autophagy” was first used by Christian De Duve in 1974 during his studies on lysosome function (1).

Morphological studies suggested that the process of autophagy has five stages: initiation, elongation, closure (three steps in autophagosome formation), maturation, and degradation (2, 3). From the early stages of their formation, autophagic structures display a unique double membrane. The phagophore, defined as an autophagosome precursor having membranes conjugated with LC3-family protein members, expands around its designated substrates and subsequently closes to become an autophagosome. In principle, phagophores may either form as isolated structures in the cytoplasm (a very unlikely possibility) or be a derivative of a specific compartment (and may then acquire constituents from other organelles by vesicles, *e*.*g*., to enable autophagosome maturation/expansion). The mature autophagosomes detach from their formation site and fuse with lysosomes to form autolysosomes. Inside the autolysosomes, the contents are hydrolyzed and subsequently recycled into the cytoplasm (4).

Autophagy studies remained purely morphological and biochemical for 20 years until the first ATG (AuTophaGy-related) genes orchestrating autophagosome biogenesis were discovered in yeast by Yoshinori Ohsumi (5). These yeast genes have functionally equivalent orthologs in mammalian cells (6, 7). Many of the ATG proteins are part of complexes that regulate autophagosome formation, including the ULK1 kinase complex (8), the Beclin-1–VPS34 class III PI3K complex (9), WIPI2–RAB11A complex (mammalian) (10), Atg5–Atg12–Atg16 complex (11), Atg9–Atg2–Atg18 complex (ATG9–ATG2–WIPI4 in mammals), and Atg8–LC3–GABARAP conjugation systems (12, 13, 14). Among the ATG proteins, the ATG8 family consists of 6 members divided into the LC3 (A, B, and C) and GABARAP (GABARAP, GABARAPL1, and GABARAPL2) subfamilies (15, 16).

The mammalian ULK1 complex functions as the most upstream component among the core protein complexes responsible for autophagosome formation. It corresponds to the yeast Atg1 complex, whose assembly serves as the primary signal initiating starvation-induced autophagy in yeast (17). The ULK1 complex comprises four proteins: the ULK1/2 kinases (homologs), ATG13, ATG101, and FIP200 (8, 9, 10, 11). FIP200 is recruited by autophagic cargo receptors to mark sites of autophagy initiation and scaffolds the assembly of the ULK1 complex (18).

Human VPS34 forms two heterotetrameric core complexes—complexes I and II. Complex I is composed of VPS34, VPS15, Beclin-1, and ATG14L, whereas complex II contains UVRAG instead of ATG14L. The presence of ATG14L or UVRAG determines how and where the two complexes are active. Both complexes produce the lipid phosphatidylinositol-3-phosphate (PI(3)P). Complex I acts at the early stages of autophagosome formation and also regulates autophagosome closure (9, 19), whereas complex II has a role in endocytic sorting along with other cellular pathways (9). ULK1 phosphorylates Beclin-1 on Ser 14, thereby enhancing the activity of complex I (20).

The ATG5–ATG12–ATG16L1 complex is part of a set of ubiquitin-like conjugation systems regulating autophagosome biogenesis (21). Like ubiquitination, these autophagy conjugation events are regulated by a cascade of events with an E1-activating enzyme, E2-conjugating enzymes, and E3 ligases (14) but exploit ATG12 and LC3-ATG8 as ubiquitin-like proteins. In the first system, ATG12 is conjugated to ATG5 in a reaction dependent on the E1-like activating enzyme ATG7, and the E2-like enzyme, ATG10. ATG12–ATG5 binds ATG16L1 to form a complex. The second system requires ATG4 to remove the C-terminal arginine of ATG8/LC3 family members to leave an exposed C-terminal glycine that can participate in the ubiquitination-like reaction (22). The E1-like enzyme for ATG8/LC3 proteins is ATG7, and the E2-like enzyme is ATG3 (23). Then, the ATG5–ATG12–ATGL16 complex acts as the E3-like ligase that conjugates ATG8/LC3 to the lipid phosphatidylethanolamine (PE) in the membranes of forming autophagosomes, a defining event in autophagosome formation. In mammalian systems, WIPI2 binds both PI(3)P and RAB11A on the recycling endosome (RE) in a coincident detection manner (24). As WIPI2 binds ATG16L1, the association of WIPI2 to the RE enables the recruitment of the ATG5–ATG12–ATG16L1 complex to this compartment (24, 25) and LC3 family member conjugation to PE in these membranes (26).

Assembly of the ATG9–ATG2–WIPI4 complex enables import of lipids to expanding, newly formed, autophagic structures through ATG2 acting as a lipid channel between the endoplasmic reticulum (ER) and phagophores (10). The formation of this complex is essential for autophagosome formation, functioning as a molecular bridge that allows lipids to be directly and efficiently delivered to the growing phagophore membrane. This complex serves as a conduit linking the ER or other lipid-donating membranes to the developing phagophore, promoting swift lipid transfer required for membrane expansion.

The discovery of ATG gene/proteins was transformative and allowed the autophagy field to move from morphological and biochemical approaches to more complex genetic and molecular studies that enabled the discoveries of multifaceted mechanisms regulating autophagosome formation. The availability of many autophagy genes/proteins led to a greater focus on trying to understand how the autophagy proteins acted as machinery and maybe less attention to understanding how cells can manage to form double-membraned vesicles in the cytoplasm. Indeed, after 30 years there is still some confusion about the compartments that give rise to autophagosomes. In this review, we will focus on studies aiming to describe the origins and shapes of autophagic precursors and how these evolve. We will not focus on the machinery, enzymology, or protein structural biology, which have been covered extensively elsewhere (27, 28, 29).

## Where does the autophagosome form?

Since autophagy substrates are found throughout the cytoplasm, one would expect that the compartment where the autophagic structures initiate must be broadly localized (30). The ER is one of the largest organelles, spreading all over the cytoplasm, and is the major site of protein synthesis and transport, protein folding, and lipid and steroid synthesis (31). The initiation membranes that are thought to be the first structures destined to form autophagosomes have been described to emerge from the ER or from “a compartment in close contact with the ER” (32, 33). For example, tomographic reconstructions of Ylä-Anttila *et al*. (34) revealed connections between autophagic structures and closely located ER cisternae. These connections were typically formed by narrow extensions from the phagophore/autophagosome to the ER. Similarly, Hayashi-Nishino *et al*. (33), using EM, showed that in mammalian cells, the ER associates with autophagic precursors and that the ER–autophagic complex appears as a subdomain of the ER. However, the literature supporting a role for the ER has not shown if phagophores are actually formed from ER membranes or whether the ER and nascent phagophores are simply in very close proximity or bridged by various proteins (32, 34, 35, 36).

In parallel with data suggesting an important role for the ER in autophagosome formation, there has been an increasing appreciation that membrane trafficking from other destinations in the cell is required for this process. For example, our early studies observed that RAB5 acts at an early stage of autophagosome formation (37). As RAB5 is a marker of the early endosome (38), we considered whether trafficking through the endocytic pathway could be important for autophagosome formation. An important clue then emerged when proteomics analysis revealed clathrin and AP2 as interactors with ATG16L1 (39). AP2 is an adaptor complex that acts on the plasma membrane to internalize cargo *via* clathrin-mediated endocytosis (40). Indeed, ATG16L1 traffics from the plasma membrane to the sites of the autophagosome formation *via* an unconventional early endosome-negative pathway, and impairment of this pathway severely compromises autophagosome formation (39).

Interestingly, ATG16L1 localizes to tubular–vesicular structures frequently found in front of the Golgi (as determined by immuno-EM) (39). A few years later, we and then others found that another ATG protein, ATG9, traffics from the plasma membrane in clathrin/AP2-positive vesicles to the sites of autophagosome formation (41, 42, 43). The trafficking of ATG9 to the RE is important for autophagosome formation (41, 42, 43). Indeed, ATG9 is cotrafficked with the transferrin receptor (TfR) from the plasma membrane to the REs *via* the classical endocytic route going from the early endosome to the RE (44, 45). The transfer to the RE and the fusion of ATG9 vesicles with those containing ATG16L1 in this compartment is soluble *N*-ethylmaleimide-sensitive factor attachment protein receptor (SNARE)-dependent. Indeed, inhibition of the SNARE VAMP3 blocks the fusion of ATG9- and ATG16L1-containing vesicles and subsequent autophagosome formation (41).

For a long time, it was assumed that REs were just an intermediate compartment where vesicles containing autophagic proteins, as ATG9 and ATG16L1, met and fused, and then these moved together to the site of the autophagosome formation (41, 46). However, we subsequently observed that the autophagic protein WIPI2 contained a sequence resembling RAB11-binding domains (similar to those seen in the RAB11FIPs, a family of RAB11-interacting proteins) (47). We then showed that RAB11A bound WIPI2 and that mutations of this RAB11-binding domain compromised the recruitment of WIPI2 to the RE and autophagosome formation (24). Importantly, WIPI2 depletion dramatically decreased the numbers of phagophores (ATG16L1-positive structures) and autophagosomes (LC3-positive structures) and caused a large increase in autophagy substrate levels. These alterations were completely rescued by overexpression of wildtype WIPI2 in WIPI2-depleted cells, but no effect was seen with a WIPI2 mutant that could not bind RAB11A properly, suggesting that most, if not all LC3, involved in autophagy is conjugated to the RAB11A compartment (24).

**Figure 1:**
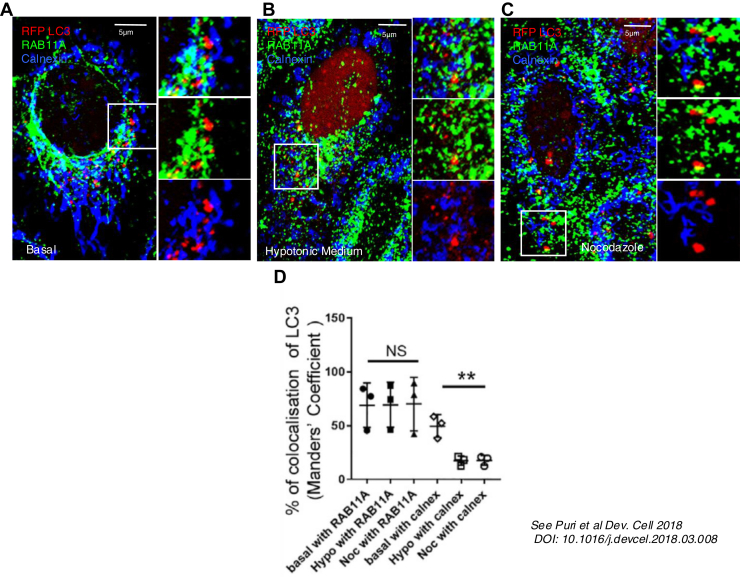
**Microtubule disruption maintains the association of LC3 with RAB11A**. *A*, under steady-state conditions, the RAB11A-positive compartment (*green*) and the calnexin-positive compartment (*blue*) are closely associated, making it difficult to determine with which compartment LC3 (*red*) is primarily associated. Disruption of microtubules by hypotonic treatment (1:3 full medium:water for 5 min) *B*, or by nocodazole (25 μM for 2 h)*C*, separates the two compartments and reveals that LC3 is predominantly associated with the RAB11A-positive compartment (*green*). *D*, quantification of colocalization between LC3 and RAB11A or calnexin under basal conditions and following hypotonic or nocodazole treatment. Figure from Puri *et al*., 2018 (24).

Key proteins associated with autophagosome biogenesis (WIPI2, DFCP1, Beclin-1, VPS34, and LC3) colocalized with the RE (RAB11A compartment). Under normal conditions, these proteins also colocalized with calnexin at ER/ER–mitochondrial contact sites. As these compartments may be so close to each other that it is impossible to discern whether the colocalization is a true interaction or simply a function of proximity, we disrupted the cytoskeleton (with nocodazole or with hypotonic medium for 5 min) and showed that the association of these proteins (WIPI2, DFCP1, Beclin-1, VPS34, and LC3) with the ER was dramatically decreased, whereas their association with the RAB11A compartment was unaltered. Note that LC3 is conjugated to the phagophore membrane so should not be disturbed by such treatments (Fig. 1). The association of LC3 with the RAB11A compartment was confirmed in multiple cell lines and primary neurons by traditional confocal microscopy, super-resolution structured illumination microscopy, and biochemical fractionation (24, 48, 49, 50, 51, 52). The roles of the RAB11 recycling compartment in autophagosome biogenesis are supported by numerous studies, particularly those from Sharon Tooze’s laboratory (46, 53, 54).

One prediction of this model is that the TfR should be within both the inner and outer membranes of nascent autophagosomes, as this transmembrane protein is trafficked from the plasma membrane to REs, from which it can recycle back to the plasma membrane. Indeed, Tooze’s group and ourselves observed the TfR in phagophore membranes (24, 46, 55) and our data suggest that the extracellular domain of the receptor is found between the two membranes of the autophagosomes. Interestingly, in these studies, transferrin or anti-TfR antibodies were introduced extracellularly. Therefore, the transferrin–TfR detected inside the autophagosome double membrane cannot be the neosynthesized protein still present in the ER, as no trafficking from the plasma membrane back to the ER has been described. Moreover, the timings of these experiments are consistent with endocytic pathway trafficking. The presence of transferrin–TfR inside the double membrane of the autophagosome provides further support that this is very likely the compartment from which the autophagosome forms. This unconventional trafficking of transmembrane proteins from the plasma membrane to autophagosome membranes is not unique to the TfR. Other plasma membrane transmembrane proteins follow a similar itinerary, including CCR5 (a G-protein–coupled receptor) (56) and likely Notch (57). As far as we are aware, there are no studies that have aimed to catalog which plasma membrane proteins are subject to this unconventional trafficking route, although we suspect that this may be relevant to most, if not all, transmembrane proteins that traffic from the plasma membrane to the RE.

**Figure 2:**
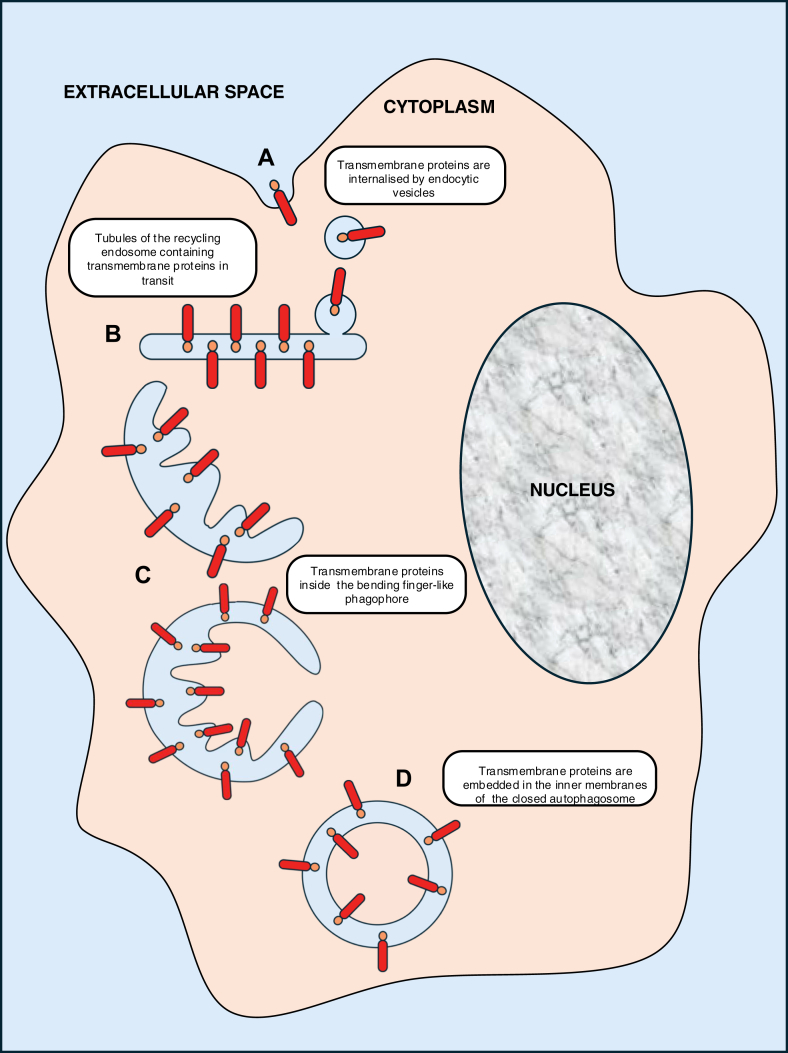
**Schematic diagram illustrating how a transmembrane protein is recruited into the autophagosome and degraded by autophagy**. *A*, transmembrane proteins are internalized by endocytic vesicles. *B*, tubules of the recycling endosome containing transmembrane proteins in transit. *C*, transmembrane proteins inside the bending finger-like phagophore. *D*, transmembrane proteins are embedded in the inner and outer membranes of the closed autophagosome.

Since the inner autophagosome membrane is degraded after fusion with lysosomes, our data suggested that the TfR should be degraded by autophagy. We confirmed this possibility, which represents an unconventional mode of degradation, as typical autophagic substrates are free in the cytoplasm and not part of the autophagic membranes themselves (58). Subsequently, we have demonstrated this phenomenon for another transmembrane protein, the chemokine receptor CCR5 (59) (Fig. 2).

We found that conventional cytoplasmic selective autophagic substrates, such as p62 (SQSTM1), are associated with LC3–RAB11A-positive membranes. Moreover, when mitochondria are damaged, autophagosomes form locally at their surfaces to generate mitophagosomes, and the organelles rapidly become rounded and completely surrounded by the RAB11A compartment, as previously reported (24). Similarly, mutant huntingtin forms cytoplasmic aggregates that are positive for both LC3 and RAB11A, suggesting that aggrephagy may also initiate from the RAB11A compartment. This interpretation is further supported by evidence that the WIPI2 mutant YI120FE, which is unable to bind RAB11A, exhibits defective aggrephagy and leads to the accumulation of mutant huntingtin aggregates (24).

Our data suggest that autophagosomes form from the RE in the cell types we have studied (from HeLa cells to neurons and that this process is relevant to bulk autophagy as well as at least some forms of selective autophagy (mitophagy and aggrephagy). Nevertheless, we cannot exclude the possibility that the RE origin may be less relevant for certain types of selective autophagy or in mammalian cell types that we have not yet examined.

## Strong contribution of the ER to the autophagosome formation

While the ER may not be the primary platform from which autophagosomes form, it plays important roles in autophagosome biogenesis. The previous suggestions that the ER is the platform from which autophagosomes emerge are likely because the autophagosome formation sites on the REs are in very close proximity (and are connected by intraorganellar contact sites) with the ER (24). Indeed, numerous articles have reported this phenomenon, whereas none have directly shown that autophagosome double membranes emanate from the ER. For example, the study from Yoshimori’s laboratory stated: “Electron tomography revealed that the ER–inner membrane complex appears as a subdomain of the ER that formed a cradle encircling the inner membrane and showed that both ER and isolation membranes are interconnected.” Indeed, Eskelinen’s laboratory, who also identified that the ER and forming autophagosomes were in close proximity, connected by membranous contacts in tomography studies, suggested “that their data raised the possibility that phagophores were formed by some kind of transformation from an existing membrane such as ER.” But they also suggested an alternative model where “one could propose that the connections between the phagophore and rough ER might be an indication of direct lipid or membrane transport from the ER to the phagophore” (34). The most recent study from Eskelinen’s group using correlative light-EM reports (60): “These findings suggest that phagophores emerge next to the ER…Initially, the phagophore precursor forms a single MCS with the ER, but as the phagophore expands, it can form additional MCSs with the ER and other organelles, which might correspond to multiple lipid-transfer sites to the elongating phagophore.” We now know that this is the case: the ATG2–WIPI4 complex tethers the ER to preautophagosomal membranes, enabling lipid transfer to nascent phagophores (61, 62). ATG9, an RE- (41, 63) and phagophore-associated protein, also binds ATG2, and ATG9 acts as a lipid scramblase equilibrating lipid levels in the phagophore membrane, thereby promoting its expansion (10, 63). On the ER side, there are two other lipid scramblases, VMP1 and TMEM41B, that appear to have an equivalent role in equilibrating levels of newly synthesized lipids over the ER membrane (64). Furthermore, ATG3, which facilitates the conjugation of LC3 to PE, localizes to this compartment (24). Thus, the arguments suggesting that ER is the platform from which autophagosomes emerge may be a consequence of the ER frequently being in close proximity with the RE (24).

### Differences and similarity of autophagosome formation between yeast and mammalian cells

In yeast, the system may differ, as its membrane trafficking system consists of the secretory and endocytic pathways, which communicate by transport to and from the *trans*-Golgi network (TGN) (65). In mammalian cells, the endocytic pathway includes early, late, and REs (66), whereas in yeast, the TGN also serves as an early endosome and RE, acting as the primary sorting station for both biosynthetic and endocytic traffic (65). In yeast, after endocytosis, cargo first accumulates in specific TGN subdomains marked by the SNARE proteins Tlg1p/Tlg2p, from which it can either be recycled back to the plasma membrane or forwarded to a prevacuolar endosome, which corresponds functionally to late endosomes or multivesicular bodies in mammalian cells and typically resides in close proximity to the vacuole, the lysosomal equivalent (67, 68). Live-cell imaging and genetic studies have shown that efficient transfer of cargo from these early/sorting TGN regions to the prevacuolar endosome depends on clathrin adaptors and Rab5-like GTPases, underscoring the tight coupling between secretory and endocytic routes in yeast (67). Moreover, a substantial fraction of endocytosed membrane proteins and lipids is recycled from the TGN back to the cell surface *via* dedicated recycling routes, reinforcing the view that the yeast TGN functionally substitutes for the early endosomes and REs of higher eukaryotes (65, 69). Consistent with its dynamic sorting role, the TGN in budding yeast, like many other endomembrane organelles, appears as multiple mobile puncta by immunofluorescence and live-cell microscopy (70, 71).

The core ATG machinery is conserved between yeast and mammals (72). However, yeast forms only a single autophagosome precursor (the preautophagosomal structure) at a time (73, 74), located near the vacuole (the yeast lysosome) (73). In contrast, in mammalian cells, autophagosome formation occurs at multiple sites, primarily in proximity to ER–RE contact regions (24, 34). In yeast, the contribution of different subcellular compartments is limited, whereas in mammalian cells, the forming autophagosome receives membrane input from multiple sources, including the ER, mitochondria, ER–Golgi intermediate compartment, endosomes, and the plasma membrane (75, 76, 77, 78, 79). PI(3)P production is essential in both systems; however, in yeast, it is synthesized predominantly on the luminal leaflet, whereas in mammals, it is generated on the cytoplasmic leaflet (80). Finally, autophagosomes in yeast are mostly spherical, whereas in mammalian cells, they display heterogeneous morphologies, often adopting elongated or finger-like shapes (19, 55, 81).

In summary, autophagosome formation in yeast and mammals not only shares some similarities but also exhibits major differences, largely because of differences in their membrane trafficking systems. As mentioned earlier, yeast lack a well-defined RE compartment, which we (and others (24, 43, 46, 48, 82, 83)) have shown to play a key role in autophagosome formation and maturation in mammalian cells.

## The shape of the forming autophagosome

### Yeast: The shape of a forming autophagosome, biology, and autophagy

For a long time, autophagy was primarily analyzed morphologically by EM through the examination of autophagosomes and autophagolysosomes in ultrathin sections (1). Oshumi in 1995 (84) used the freeze–replica method to characterize the morphology of yeast autophagosomes. Recent studies using more advanced techniques have revealed details of the structure–shape of a forming autophagosome in yeast. Using correlative cryo-electron tomography, Bieber *et al*. (85) captured the key steps of autophagosome biogenesis, including early phagophores, which have a disk shape that is slightly bent to form a small concave structure. This concave structure expands into a cup-shaped phagophore with a clearly visible opening to the cytoplasm. The autophagosome closes when the rim is not visible any more. Phagophores and autophagosomes have similar sizes. Closed autophagosomes are almost perfectly spherical, whereas phagophores are more elongated. The autophagosome expansion does not appear to require vesicular traffic from other compartments but mostly involves lipid transfer from the ER, as is seen in mammalian cells (85).

### The shape of forming autophagosomes in mammalian cells

The shape of forming mammalian autophagosomes has been extensively examined using correlative-EM tomography (34). The partial reconstruction of an autophagic structure (one half of the structure) revealed a cup shape with gaps in the limiting membrane (34). Similarly, Gudmundsson *et al*. (60), using correlative EM, observed that phagophore precursors emerge next to the ER as bud-like highly curved membrane cisternae with a small opening to the cytosol. The phagophore precursors then open to form more flat cisternae that elongate and curve to form the classically described crescent-shaped phagophores. The phagophore had elongated shapes until closure. Li *et al.* investigated autophagosome formation during xenophagy, the autophagic degradation of internalized bacteria (reviewed in 86). Their data reveal that the autophagic structures around *Salmonella* have a disk shape that expands to cup-shaped phagophores (87).

Sakai *et al*. (88) built a theoretical model that integrated the membrane morphological change and entropic partitioning of putative curvature generators, which they used to investigate the autophagosome formation process quantitatively. Subsequently, the same group (89) used an *in vivo* approach to determine the shape of forming autophagosomes using three-dimensional electron micrographs of more than 100 phagophores. They found that the phagophore is longitudinally elongated, and the rim has an outwardly recurved shape (89).

Analyses of autophagosome structure by correlative EM have some limitations. It is not possible to conclude if it is an open or closed autophagosome because the tomogram only includes 500 nm of a section, and mammalian autophagosomes are larger. Indeed, early autophagic structures can be quite large (from 500 to 1500 nm) (55). Furthermore, it is quite difficult to unambiguously identify structures using only a subset of membranes within thick sections containing a plethora of other endocytic and biosynthetic vesicles and tubules using only morphological criteria in the absence of molecular markers.

**Figure 3:**
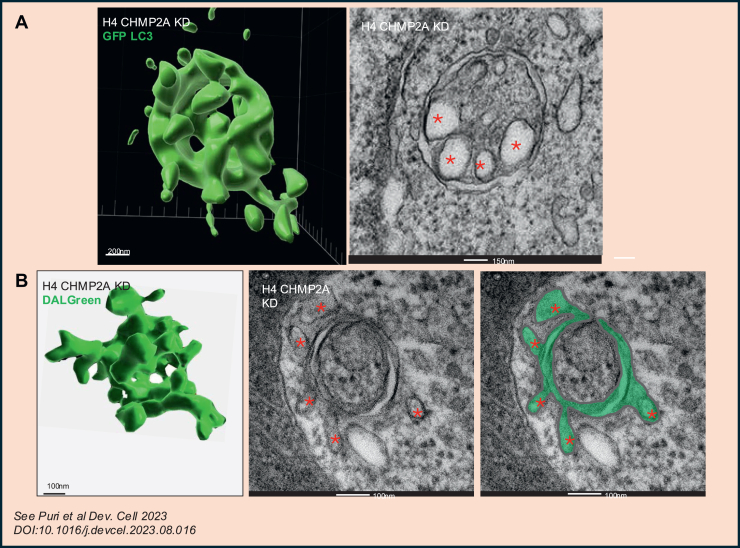
**Super-resolution microscopy images resemble 2D EM images**. *A* and *B*, H4 cells were treated with CHMP2A siRNAs for 3 days. After 2 h of starvation, some cells were fixed and processed for conventional EM (*right panels*; *red asterisks* indicate likely finger-like structures in the phagophore). Note the resemblance between the structures observed by super-resolution microscopy (SIM) using different autophagosome markers (LC3 or DALGreen; *left panels*) and those observed by conventional EM. See Puri *et al*. (55).

We recently used structured illumination super-resolution microscopy (SIM) and advanced SIM^2^ super-resolution live microscopy to clarify the structure of forming autophagosomes with a detail and resolution close to what is possible using EM. We studied normal cells and also blocked the closure of the forming autophagosome by inhibiting the endosomal sorting complex required for transport (ESCRT) complex (that is required for phagophore closure (90, 91)). Using this approach, we showed that the morphology of a phagophore was more complicated than was considered previously (60, 85, 89, 92). Surprisingly, phagophores have finger-like structures, instead of being cisternae with a single opening. By using SIM^2^ that allows super-resolution definition with live-cell imaging, we observed that finger-like phagophores are highly dynamic structures that evolve into more characteristic spherical closed autophagosomes. The finger-like phagophore works like a hand that closes in a fist as it captures its cargoes (55). With these SIM data in mind, we were able to reconcile such structures with our electron microscope images of phagophores after being sensitized to their finger-like structures (55) (Fig. 3). This finger-like model of phagophores may not necessarily conflict with previous data suggesting that phagophores are pseudospherical with single openings if there are such structures among late-stage phagophores prior to their final closure. The finger-like precursors were likely overlooked by previous EM studies because they were unexpected and very difficult to reconstruct from tomograms given their size and complex morphologies—the finger-like autophagosome precursor structures occupy larger volumes than the 300 to 500 nm typically included in tomograms (93). Interestingly, the finger-like phagophore structures with multiple apertures that we observed by SIM microscopy and whole-mount EM also appear in 3D-rendered surface model reconstructions of super-resolved z-stack images of LC3B autophagosomes (43, 81).

## How do phagophores close into an autophagosome

**Figure 4:**
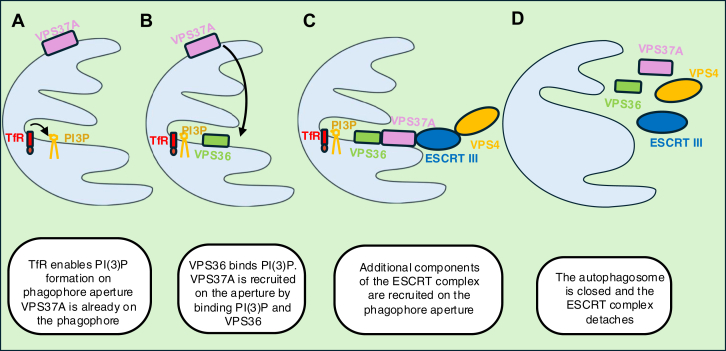
**Schematic diagram illustrating how transferrin receptor (TfR) regulates autophagosome closure**. *A*, TfR enables PI(3)P formation on phagophore aperture VPS37A is already on the phagophore. *B*, VPS36 binds PI(3)P. VPS37A is recruited on the aperture by binding PI(3)P and VPS36. *C*, additional components of the ESCRT complex are recruited on the phagophore aperture. *D*, the autophagosome is closed, and the ESCRT complex detaches. ESCRT, endosomal sorting complex required for transport; PI(3)P, phosphatidylinositol-3-phosphate.

The process of autophagosome closure was thought to be topologically related to the biogenesis of multivesicular bodies, virus budding, and cytokinesis, where membrane remodeling involves the ESCRT machinery (Fig. 4) (94). In 2018, Takahashi *et al*. (90) revealed that the ESCRT-III subunit CHMP2A and the AAA-ATPase VPS4A are essential for autophagosome closure. The ESCRT complex is divided in five subgroups: ESCRT-0, ESCRT-I, ESCRT-II, ESCRT-III, and ESCRT-associated proteins. ESCRT-0 does not appear to be important for autophagosome closure, as the first protein of the complex that is recruited on the phagophore is VPS37A (ESCRT-I) (91). Javed *et al*. (70) demonstrated that the ESCRT-I protein, VPS37A, interacts with several ATG8 family members—specifically GABARAP and LC3A but not LC3B. Notably, depletion of VPS37A does not prevent the recruitment of VPS36 (an ESCRT-II component) to the phagophore. Since VPS36 (ESCRT-II) is a PI(3)P-binding protein, this finding raises important questions about the role of PI(3)P in autophagosome closure—PI(3)P facilitates the assembly and activation of the ESCRT machinery in other settings (71). Interestingly, PI(3)P depletion caused VPS37A to distribute diffusely across the phagophore membrane, rather than localizing to the apertures. This suggests that while VPS37A may be the first ESCRT protein recruited to the autophagosome, its specific localization to the phagophore aperture requires both PI(3)P and VPS36, consistent with a model of coincident detection. These data can explain why PI(3)P is required for autophagosome closure, in addition to its conventional role upstream of LC3-II formation (19). Interestingly, the PI(3)P for both these steps is generated by the VPS34 complex I–containing proteins, including VPS34, Beclin-1, and ATG14. Depletion of PI(3)P or VPS36 results in the accumulation of unclosed, finger-like phagophores (Fig. 4).

The VPS34 complex is recruited to the RAB11A/RE *via* the interaction of its constituent proteins and the TfR (which traffics to this compartment, as described above) (Fig. 4). This interaction requires ubiquitination of the TfR cytoplasmic domain by MARCH8 (an E3 ubiquitin ligase committed to targeted protein degradation) enabled by the dissociation of iron from transferrin bound to its receptor. Indeed, this is a noncanonical function of the receptor that is independent of transferrin binding (19).

## From “autophagodome” to free autophagosome: How do autophagic structures detach from the RE?

**Figure 5:**
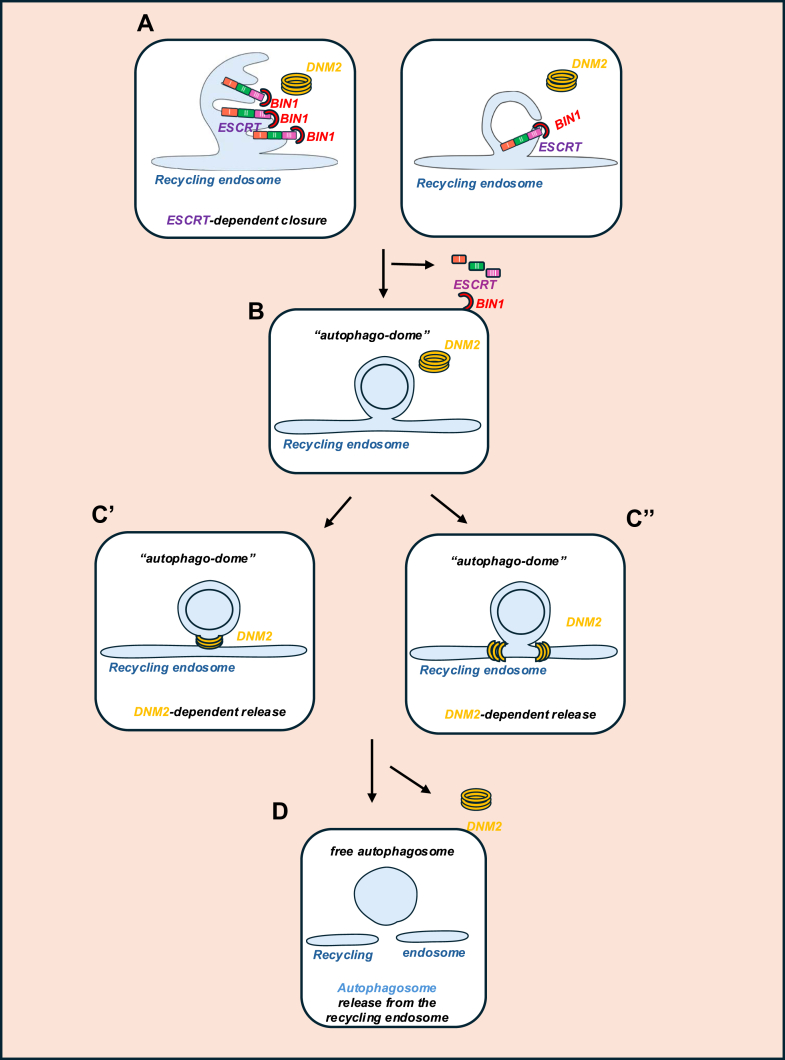
**Model of the antagonistic control of autophagosome release from the recycling endosome by BIN1 and DNM2**. *A*, in unclosed autophagosomes, ESCRT components bind BIN1, which prevents DNM2 from interacting with LC3 and also inhibits DNM2 activity. *B*, after an autophagosome is closed, the ESCRT complex is disassembled. BIN1 is not on autophagosomes, allowing active DNM2 to be recruited to autophagosome membranes by binding LC3. *C*, DNM2 mediates the release of the autophagosome from the recycling endosome, which subsequently becomes fragmented. *C′*, DNM2 scission occurs at the neck of the autophagosome. *C”*, DNM2 scission occurs laterally at its point of connection with the recycling endosome. *D*, autophagosome release from the recycling endosome. DNM2, dynamin-2; ESCRT, endosomal sorting complex required for transport.

How autophagic structures detach from the platform where they are assembled is a question that has not been frequently considered—indeed, this release process has been overlooked in the conventional steps of the autophagy itinerary in the literature. We have shown that the closure of an autophagosome is a prerequisite for its detachment from the RE platform where it was generated (42) and that the release step is required for subsequent autophagosome–lysosome fusion (19, 95). This observation has two important implications: (1) open structures (phagophores) are not found freely in the cytoplasm and (2) there is a transient intermediate closed autophagic structure with its captured contents still attached to the RE platform (Fig. 5). As this structure was previously undescribed, we thought it deserved a name, which we have coined as an “autophagodome” (96).

This scission of autophagodomes from the RE to form autophagosomes is mediated by dynamin-2 (DNM2), which is recruited to closed autophagodomes by binding LC3B through an LC3-interacting region. DNM2 is mutated in centronuclear myopathy, causing the accumulation of closed autophagosomes still attached to the RE, which results in impaired autophagy flux in cells from patients or a knock-in mouse model of this disease (97). Although we know that DNM2 is responsible for autophagosome release, the underlying mechanism remains unclear. One possibility is that, once the ESCRT complex seals the double membrane, the only membrane that needs to be severed is the one connecting the autophagosome to the RE. However, it is not known whether the scission occurs at the neck of the autophagosome or laterally at its point of connection with the RE (Fig. 5). Both models are consistent with the established function of DNM2 in severing a single lipid bilayer. In contrast, it is highly unlikely that DNM2 mediates scission of a double bilayer (see model in Fig. 5).

The mechanism underlying the requirement for autophagosome closure prior to their release from the RE membranes was recently found to be enabled by BIN1. Before autophagosomes are closed, BIN1, an inhibitor of DNM2, is sequestered to these structures by interacting with ESCRT-III. After closure, the ESCRT machinery disassembles, BIN1 is released, and DNM2 can then act to release the autophagosomes from the RE (95) (Fig. 5). This process likely occurs in diverse cell types.

BIN1 is a major Alzheimer’s disease risk gene (98) and analyses suggest that the risk allele is associated with increased microglial expression, since the relevant SNP lies in a microglial-specific enhancer that regulates BIN1 expression in these cells but not in neurons or astrocytes (98, 99, 100). BIN1 overexpression in microglia (and other cells) impairs autophagy flux by compromising DNM2-dependent autophagosome release from the RE (95). This may be an important defect underlying Alzheimer’s pathogenesis, as microglial-specific autophagy compromise makes these cells more prone to senescence and neuroinflammation, while compromising their ability to remove amyloid-beta in an Alzheimer’s disease mouse model (101, 102).

## Conclusions and open questions

**Figure 6:**
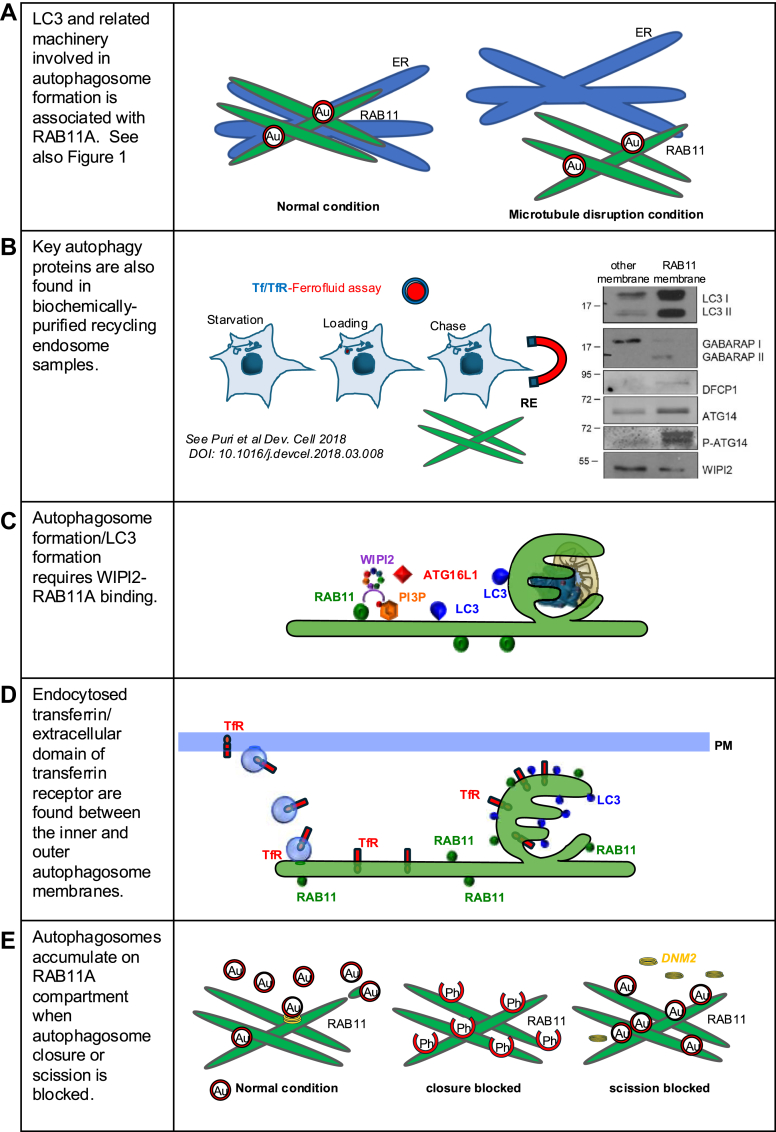
**Major arguments supporting the RAB11A compartment/recycling endosome as the source of mammalian autophagosomes**. *A*, LC3 and related machinery involved in autophagosome formation is associated with RAB11A. This association is robust to microtubule disruption, whereas the association of these autophagy components with the ER is significantly reduced by microtubule disruption. *B*, key autophagy proteins are also found in biochemically purified recycling endosome samples. HeLa cells were starved for 1 h, loaded with ferrofluid–Tf or TfR antibody for 1 h and chased for 15 min in starvation medium. The cells were then fragmented, and the membranes containing ferrofluid–Tf or TfR antibody (RAB11A membrane) or not containing ferrofluid–Tf or TfR antibody (other membrane) were separated and processed for immunoblotting. The blots show autophagic proteins, such as LC3, GABARAP (ATG8 family) DFCP1, ATG14, and WIPI2 (PI(3)P-binding proteins localized mainly in RAB11-positive membrane). *C*, autophagosome formation/LC3 formation requires WIPI2–RAB11A binding. *D*, endocytosed transferrin/extracellular domain of TfR are found between the inner and outer autophagosome membranes. *E*, autophagosomes accumulate on the RAB11A compartment when autophagosome closure or scission is blocked. ER, endoplasmic reticulum; PI(3)P, phosphatidylinositol-3-phosphate; TfR, transferrin receptor.

Autophagosome formation is closely linked to the endocytic pathway, sharing several proteins, protein complexes, and subcellular compartments. Several lines of evidence show that the RE is the major platform where autophagic structures form (Fig. 6). Importantly, this does not exclude the involvement of other organelles, such as the ER, which play critical roles in autophagosome development and maturation by shuttling lipids to nascent autophagosomes *via* ATG2.

Despite these advances, many questions remain unanswered and warrant further investigation in the coming years, especially given the importance of this pathway in diseases, such as cancer and neurodegeneration. For example, it is unclear how the finger-like outgrowths from the RE are shaped and how they are closed by the ESCRT complex.

## Conflict of interest

D. C. R. is a consultant for Drishti Discoveries, PAQ Therapeutics, MindRank AI, Retro Biosciences, Alexion Pharma International Operations Limited, Carlyle Investment Management LLC, Aladdin Healthcare Technologies Ltd, Nido Biosciences, ProtosBio, and is a cofounder of Acuity Technologies Ltd. C. P. declares no conflicts of interest with the contents of this article.

## References

[1] De Duve C. (1966). The significance of lysosomes in pathology and medicine. Proc. Inst. Med. Chic.

[2] Kimura S., Noda T., Yoshimori T. (2007). Dissection of the autophagosome maturation process by a novel reporter protein, tandem fluorescent-tagged LC3. Autophagy.

[3] Chen D., Fan W., Lu Y., Ding X., Chen S., Zhong Q. (2012). A mammalian autophagosome maturation mechanism mediated by TECPR1 and the Atg12-Atg5 conjugate. Mol. Cell.

[4] Zhou C., Wu Z., Du W., Que H., Wang Y., Ouyang Q. (2022). Recycling of autophagosomal components from autolysosomes by the recycler complex. Nat. Cell Biol..

[5] Tsukada M., Ohsumi Y. (1993). Isolation and characterization of autophagy-defective mutants of Saccharomyces cerevisiae. FEBS Lett..

[6] Nakatogawa H., Suzuki K., Kamada Y., Ohsumi Y. (2009). Dynamics and diversity in autophagy mechanisms: lessons from yeast. Nat. Rev. Mol. Cell Biol..

[7] Klionsky D.J., Cregg J.M., Dunn W.A., Emr S.D., Sakai Y., Sandoval I.V. (2003). A unified nomenclature for yeast autophagy-related genes. Dev. Cell.

[8] Karanasios E., Walker S.A., Okkenhaug H., Manifava M., Hummel E., Zimmermann H. (2016). Autophagy initiation by ULK complex assembly on ER tubulovesicular regions marked by ATG9 vesicles. Nat. Commun..

[9] Ohashi Y., Tremel S., Williams R.L. (2019). VPS34 complexes from a structural perspective. J. Lipid Res..

[10] Gomez-Sanchez R., Rose J., Guimaraes R., Mari M., Papinski D., Rieter E. (2018). Atg9 establishes Atg2-dependent contact sites between the endoplasmic reticulum and phagophores. J. Cell Biol..

[11] Walczak M., Martens S. (2013). Dissecting the role of the Atg12-Atg5-Atg16 complex during autophagosome formation. Autophagy.

[12] Mizushima N. (2020). The ATG conjugation systems in autophagy. Curr. Opin. Cell Biol..

[13] Tsuboyama K., Koyama-Honda I., Sakamaki Y., Koike M., Morishita H., Mizushima N. (2016). The ATG conjugation systems are important for degradation of the inner autophagosomal membrane. Science.

[14] Ohsumi Y., Mizushima N. (2004). Two ubiquitin-like conjugation systems essential for autophagy. Semin. Cell Dev. Biol..

[15] Kabeya Y., Mizushima N., Ueno T., Yamamoto A., Kirisako T., Noda T. (2000). LC3, a mammalian homologue of yeast Apg8p, is localized in autophagosome membranes after processing. EMBO J..

[16] Kabeya Y., Mizushima N., Yamamoto A., Oshitani-Okamoto S., Ohsumi Y., Yoshimori T. (2004). LC3, GABARAP and GATE16 localize to autophagosomal membrane depending on form-II formation. J. Cell Sci..

[17] Wong P.M., Puente C., Ganley I.G., Jiang X. (2013). The ULK1 complex: sensing nutrient signals for autophagy activation. Autophagy.

[18] Shi X., Yokom A.L., Wang C., Young L.N., Youle R.J., Hurley J.H. (2020). ULK complex organization in autophagy by a C-shaped FIP200 N-terminal domain dimer. J. Cell Biol..

[19] Puri C., Park S.J., Wrobel L., Rubinsztein D.C. (2025). Transferrin receptor controls both autophagosome formation and closure via phosphatidylinositol 3-phosphate synthesis. Dev. Cell.

[20] Russell R.C., Tian Y., Yuan H., Park H.W., Chang Y.Y., Kim J. (2013). ULK1 induces autophagy by phosphorylating Beclin-1 and activating VPS34 lipid kinase. Nat. Cell Biol..

[21] Cappadocia L., Lima C.D. (2018). Ubiquitin-like protein conjugation: structures, chemistry, and mechanism. Chem. Rev..

[22] Fujita N., Hayashi-Nishino M., Fukumoto H., Omori H., Yamamoto A., Noda T. (2008). An Atg4B mutant hampers the lipidation of LC3 paralogues and causes defects in autophagosome closure. Mol. Biol. Cell.

[23] Metlagel Z., Otomo C., Takaesu G., Otomo T. (2013). Structural basis of ATG3 recognition by the autophagic ubiquitin-like protein ATG12. Proc. Natl. Acad. Sci. U. S. A..

[24] Puri C., Vicinanza M., Ashkenazi A., Gratian M.J., Zhang Q., Bento C.F. (2018). The RAB11A-Positive compartment is a primary platform for autophagosome assembly mediated by WIPI2 recognition of PI3P-RAB11A. Dev. Cell.

[25] Dooley H.C., Razi M., Polson H.E., Girardin S.E., Wilson M.I., Tooze S.A. (2014). WIPI2 links LC3 conjugation with PI3P, autophagosome formation, and pathogen clearance by recruiting Atg12-5-16L1. Mol. Cell.

[26] Lee Y.K., Lee J.A. (2016). Role of the mammalian ATG8/LC3 family in autophagy: differential and compensatory roles in the spatiotemporal regulation of autophagy. BMB Rep..

[27] Lai L.T.F., Ye H., Zhang W., Jiang L., Lau W.C.Y. (2019). Structural biology and electron microscopy of the autophagy molecular machinery. Cells.

[28] Suzuki H., Osawa T., Fujioka Y., Noda N.N. (2017). Structural biology of the core autophagy machinery. Curr. Opin. Struct. Biol..

[29] Galluzzi L., Baehrecke E.H., Ballabio A., Boya P., Bravo-San Pedro J.M., Cecconi F. (2017). Molecular definitions of autophagy and related processes. EMBO J..

[30] Graef M. (2020). Recent advances in the understanding of autophagosome biogenesis. F1000Res..

[31] Schwarz D.S., Blower M.D. (2016). The endoplasmic reticulum: structure, function and response to cellular signaling. Cell Mol. Life Sci..

[32] Hayashi-Nishino M., Fujita N., Noda T., Yamaguchi A., Yoshimori T., Yamamoto A. (2009). A subdomain of the endoplasmic reticulum forms a cradle for autophagosome formation. Nat. Cell Biol..

[33] Hayashi-Nishino M., Fujita N., Noda T., Yamaguchi A., Yoshimori T., Yamamoto A. (2010). Electron tomography reveals the endoplasmic reticulum as a membrane source for autophagosome formation. Autophagy.

[34] Yla-Anttila P., Vihinen H., Jokitalo E., Eskelinen E.L. (2009). 3D tomography reveals connections between the phagophore and endoplasmic reticulum. Autophagy.

[35] Kishi-Itakura C., Koyama-Honda I., Itakura E., Mizushima N. (2014). Ultrastructural analysis of autophagosome organization using mammalian autophagy-deficient cells. J. Cell Sci..

[36] Zhen Y., Stenmark H. (2023). Autophagosome biogenesis. Cells.

[37] Ravikumar B., Imarisio S., Sarkar S., O'Kane C.J., Rubinsztein D.C. (2008). Rab5 modulates aggregation and toxicity of mutant huntingtin through macroautophagy in cell and fly models of Huntington disease. J. Cell Sci..

[38] Bucci C., Parton R.G., Mather I.H., Stunnenberg H., Simons K., Hoflack B. (1992). The small GTPase rab5 functions as a regulatory factor in the early endocytic pathway. Cell.

[39] Ravikumar B., Moreau K., Jahreiss L., Puri C., Rubinsztein D.C. (2010). Plasma membrane contributes to the formation of pre-autophagosomal structures. Nat. Cell Biol..

[40] Collins B.M., McCoy A.J., Kent H.M., Evans P.R., Owen D.J. (2002). Molecular architecture and functional model of the endocytic AP2 complex. Cell.

[41] Puri C., Renna M., Bento C.F., Moreau K., Rubinsztein D.C. (2013). Diverse autophagosome membrane sources coalesce in recycling endosomes. Cell.

[42] Popovic D., Dikic I. (2014). TBC1D5 and the AP2 complex regulate ATG9 trafficking and initiation of autophagy. EMBO Rep..

[43] Imai K., Hao F., Fujita N., Tsuji Y., Oe Y., Araki Y. (2016). Atg9A trafficking through the recycling endosomes is required for autophagosome formation. J. Cell Sci..

[44] Mayle K.M., Le A.M., Kamei D.T. (2012). The intracellular trafficking pathway of transferrin. Biochim. Biophys. Acta..

[45] Orsi A., Razi M., Dooley H.C., Robinson D., Weston A.E., Collinson L.M. (2012). Dynamic and transient interactions of Atg9 with autophagosomes, but not membrane integration, are required for autophagy. Mol. Biol. Cell.

[46] Longatti A., Lamb C.A., Razi M., Yoshimura S., Barr F.A., Tooze S.A. (2012). TBC1D14 regulates autophagosome formation via Rab11- and ULK1-positive recycling endosomes. J. Cell Biol..

[47] Prekeris R., Davies J.M., Scheller R.H. (2001). Identification of a novel Rab11/25 binding domain present in Eferin and Rip proteins. J. Biol. Chem..

[48] Janusz-Kaminska A., Brzozowska A., Tempes A., Urbanska M., Blazejczyk M., Milek J. (2024). Rab11 regulates autophagy at dendritic spines in an mTOR- and NMDA-dependent manner. Mol. Biol. Cell.

[49] Gong Y., Hu Y., Huang J., Wang H. (2022). RAB11A aggravates PDGF-BB-stimulated proliferation, migration, and inflammation of airway smooth muscle cells via affecting the NF-kappaB and PI3K/AKT pathways. Allergol. Immunopathol..

[50] Kampf L.L., Schneider R., Gerstner L., Thunauer R., Chen M., Helmstadter M. (2019). TBC1D8B mutations implicate RAB11-Dependent vesicular trafficking in the pathogenesis of nephrotic syndrome. J. Am. Soc. Nephrol..

[51] Li D., Huang S., Zhu J., Hu T., Han Z., Zhang S. (2019). Exosomes from MiR-21-5p-Increased neurons play a role in neuroprotection by suppressing Rab11a-Mediated neuronal autophagy in vitro after traumatic brain injury. Med. Sci. Monit..

[52] Wang Y., Ren Y., Li N., Zhao J., Zhao S. (2022). Rab11a promotes the malignant progression of ovarian cancer by inducing autophagy. Genes Genomics.

[53] Lamb C.A., Nuhlen S., Judith D., Frith D., Snijders A.P., Behrends C. (2016). TBC1D14 regulates autophagy via the TRAPP complex and ATG9 traffic. EMBO J..

[54] Soreng K., Munson M.J., Lamb C.A., Bjorndal G.T., Pankiv S., Carlsson S.R. (2018). SNX18 regulates ATG9A trafficking from recycling endosomes by recruiting Dynamin-2. EMBO Rep..

[55] Puri C., Gratian M.J., Rubinsztein D.C. (2023). Mammalian autophagosomes form from finger-like phagophores. Dev. Cell.

[56] Festa B.P., Siddiqi F.H., Jimenez-Sanchez M., Rubinsztein D.C. (2024). Microglial cytokines poison neuronal autophagy via CCR5, a druggable target. Autophagy.

[57] Wu X., Fleming A., Ricketts T., Pavel M., Virgin H., Menzies F.M. (2016). Autophagy regulates notch degradation and modulates stem cell development and neurogenesis. Nat. Commun..

[58] Fleming A., Bourdenx M., Fujimaki M., Karabiyik C., Krause G.J., Lopez A. (2022). The different autophagy degradation pathways and neurodegeneration. Neuron..

[59] Festa B.P., Siddiqi F.H., Jimenez-Sanchez M., Won H., Rob M., Djajadikerta A. (2023). Microglial-to-neuronal CCR5 signaling regulates autophagy in neurodegeneration. Neuron.

[60] Gudmundsson S.R., Kallio K.A., Vihinen H., Jokitalo E., Ktistakis N., Eskelinen E.L. (2022). Morphology of phagophore precursors by correlative light-electron microscopy. Cells.

[61] Kotani T., Kirisako H., Koizumi M., Ohsumi Y., Nakatogawa H. (2018). The Atg2-Atg18 complex tethers pre-autophagosomal membranes to the endoplasmic reticulum for autophagosome formation. Proc. Natl. Acad. Sci. U. S. A..

[62] Valverde D.P., Yu S., Boggavarapu V., Kumar N., Lees J.A., Walz T. (2019). ATG2 transports lipids to promote autophagosome biogenesis. J. Cell Biol..

[63] van Vliet A.R., Chiduza G.N., Maslen S.L., Pye V.E., Joshi D., De Tito S. (2022). ATG9A and ATG2A form a heteromeric complex essential for autophagosome formation. Mol. Cell.

[64] Zhang T., Li Y.E., Yuan Y., Du X., Wang Y., Dong X. (2021). TMEM41B and VMP1 are phospholipid scramblases. Autophagy.

[65] Day K.J., Casler J.C., Glick B.S. (2018). Budding yeast has a minimal endomembrane System. Dev. Cell.

[66] Maxfield F.R., McGraw T.E. (2004). Endocytic recycling. Nat. Rev. Mol. Cell Biol..

[67] Pelham H.R. (1991). Recycling of proteins between the endoplasmic reticulum and Golgi complex. Curr. Opin. Cell Biol..

[68] Vida T.A., Emr S.D. (1995). A new vital stain for visualizing vacuolar membrane dynamics and endocytosis in yeast. J. Cell Biol..

[69] Malinska K., Malinsky J., Opekarova M., Tanner W. (2003). Visualization of protein compartmentation within the plasma membrane of living yeast cells. Mol. Biol. Cell.

[70] Bhave M., Papanikou E., Iyer P., Pandya K., Jain B.K., Ganguly A. (2014). Golgi enlargement in arf-depleted yeast cells is due to altered dynamics of cisternal maturation. J. Cell Sci.

[71] Losev E., Reinke C.A., Jellen J., Strongin D.E., Bevis B.J., Glick B.S. (2006). Golgi maturation visualized in living yeast. Nature.

[72] Delorme-Axford E., Guimaraes R.S., Reggiori F., Klionsky D.J. (2015). The yeast Saccharomyces cerevisiae: an overview of methods to study autophagy progression. Methods.

[73] Welter E., Thumm M., Krick R. (2010). Quantification of nonselective bulk autophagy in S. cerevisiae using Pgk1-GFP. Autophagy.

[74] Reggiori F., Klionsky D.J. (2013). Autophagic processes in yeast: mechanism, machinery and regulation. Genetics.

[75] Axe E.L., Walker S.A., Manifava M., Chandra P., Roderick H.L., Habermann A. (2008). Autophagosome formation from membrane compartments enriched in phosphatidylinositol 3-phosphate and dynamically connected to the endoplasmic reticulum. J. Cell Biol..

[76] Ge L., Melville D., Zhang M., Schekman R. (2013). The ER-Golgi intermediate compartment is a key membrane source for the LC3 lipidation step of autophagosome biogenesis. Elife.

[77] Ge L., Zhang M., Kenny S.J., Liu D., Maeda M., Saito K. (2017). Remodeling of ER-exit sites initiates a membrane supply pathway for autophagosome biogenesis. EMBO Rep..

[78] Mari M., Griffith J., Rieter E., Krishnappa L., Klionsky D.J., Reggiori F. (2010). An Atg9-containing compartment that functions in the early steps of autophagosome biogenesis. J. Cell Biol..

[79] Yamamoto H., Kakuta S., Watanabe T.M., Kitamura A., Sekito T., Kondo-Kakuta C. (2012). Atg9 vesicles are an important membrane source during early steps of autophagosome formation. J. Cell Biol..

[80] Cheng J., Fujita A., Yamamoto H., Tatematsu T., Kakuta S., Obara K. (2014). Yeast and mammalian autophagosomes exhibit distinct phosphatidylinositol 3-phosphate asymmetries. Nat. Commun..

[81] Das D., Sharma M., Gahlot D., Nia S.S., Gain C., Mecklenburg M. (2024). VPS4A is the selective receptor for lipophagy in mice and humans. Mol. Cell.

[82] Da Graca J., Thiola C., Rouabah M., Guerrera I.C., El Khallouki N., Romao M. (2025). ER-endosome contacts generate a local environment promoting phagophore formation. Cell Rep..

[83] Wang K., Li S., Worku T., Hao X., Yang L., Zhang S. (2017). Rab11a is required for porcine reproductive and respiratory syndrome virus induced autophagy to promote viral replication. Biochem. Biophys. Res. Commun..

[84] Baba M., Osumi M., Ohsumi Y. (1995). Analysis of the membrane structures involved in autophagy in yeast by freeze-replica method. Cell Struct. Funct..

[85] Bieber A., Capitanio C., Erdmann P.S., Fiedler F., Beck F., Lee C.W. (2022). In situ structural analysis reveals membrane shape transitions during autophagosome formation. Proc. Natl. Acad. Sci. U. S. A..

[86] Bauckman K.A., Owusu-Boaitey N., Mysorekar I.U. (2015). Selective autophagy: xenophagy. Methods.

[87] Li M., Tripathi-Giesgen I., Schulman B.A., Baumeister W., Wilfling F. (2023). In situ snapshots along a mammalian selective autophagy pathway. Proc. Natl. Acad. Sci. U. S. A..

[88] Sakai Y., Koyama-Honda I., Tachikawa M., Knorr R.L., Mizushima N. (2020). Modeling membrane morphological change during autophagosome Formation. iScience.

[89] Sakai Y., Takahashi S., Koyama-Honda I., Saito C., Mizushima N. (2024). Experimental determination and mathematical modeling of standard shapes of forming autophagosomes. Nat. Commun..

[90] Takahashi Y., He H., Tang Z., Hattori T., Liu Y., Young M.M. (2018). An autophagy assay reveals the ESCRT-III component CHMP2A as a regulator of phagophore closure. Nat. Commun..

[91] Takahashi Y., Liang X., Hattori T., Tang Z., He H., Chen H. (2019). VPS37A directs ESCRT recruitment for phagophore closure. J. Cell Biol..

[92] Uemura T., Yamamoto M., Kametaka A., Sou Y.S., Yabashi A., Yamada A. (2014). A cluster of thin tubular structures mediates transformation of the endoplasmic reticulum to autophagic isolation membrane. Mol. Cell Biol..

[93] Aoyama K., Takazaki H., Arie M., Suemune H., Kawai S. (2025). Cryo-STEM tomography for cell biology using thick lamella. Microscopy (Oxf).

[94] Jiang W., Chen X., Ji C., Zhang W., Song J., Li J. (2021). Key regulators of autophagosome closure. Cells.

[95] Palmer J.E., Puri C., O'Rourke R.S., Son S.M., Sang C., Huang Y.A. (2025). Coordination of autophagosome closure and release by the Alzheimer's disease-associated protein BIN1. Cell Rep..

[96] Puri C., Rubinsztein D.C. (2024). Mammalian phagophores with finger-like shapes emerge from recycling endosomes. Autophagy.

[97] Puri C., Manni M.M., Vicinanza M., Hilcenko C., Zhu Y., Runwal G. (2020). A DNM2 centronuclear myopathy mutation reveals a link between recycling endosome scission and Autophagy. Dev. Cell.

[98] Dourlen P., Kilinc D., Landrieu I., Chapuis J., Lambert J.C. (2025). BIN1 and Alzheimer's disease: the tau connection. Trends Neurosci..

[99] Sudwarts A., Ramesha S., Gao T., Ponnusamy M., Wang S., Hansen M. (2022). BIN1 is a key regulator of proinflammatory and neurodegeneration-related activation in microglia. Mol. Neurodegener..

[100] Sudwarts A., Thinakaran G. (2023). Alzheimer's genes in microglia: a risk worth investigating. Mol. Neurodegener..

[101] Gaikwad S., Senapati S., Haque M.A., Kayed R. (2024). Senescence, brain inflammation, and oligomeric tau drive cognitive decline in Alzheimer's disease: evidence from clinical and preclinical studies. Alzheimers Dement..

[102] Heneka M.T., Carson M.J., El Khoury J., Landreth G.E., Brosseron F., Feinstein D.L. (2015). Neuroinflammation in Alzheimer's disease. Lancet Neurol..

